# The relationship between the latent profiles of alexithymia and self-disclosure in female patients with pelvic floor dysfunction

**DOI:** 10.3389/fpsyt.2026.1941309

**Published:** 2026-09-03

**Authors:** Lu Jiang, Ru Wang, Zhengwei Luo, Ting Wu, Nian Liu, Xiaoli Guo

**Affiliations:** 1 School of Nursing, Xinjiang Medical University, Urumqi, Xinjiang, China; 2 Department of Urology, First Affiliated Hospital of Xinjiang Medical University, Urumqi, Xinjiang, China; 3 Department of Gynecology, First Affiliated Hospital of Xinjiang Medical University, Urumqi, Xinjiang, China

**Keywords:** alexithymia, female, latent profile analysis, pelvic floor dysfunction, self-disclosure

## Abstract

**Background:**

Alexithymia is common among female patients with pelvic floor dysfunction (PFD) and has been associated with negative emotions, poorer quality of life, impaired disease management, and reduced self-disclosure. This study aimed to identify latent profiles of alexithymia among female patients with PFD, examine factors associated with profile membership, and investigate the association between these profiles and self-disclosure.

**Methods:**

A cross-sectional study was conducted with 450 female patients with PFD in Urumqi, Xinjiang, China, from December 2025 to April 2026. Data were collected using a general information questionnaire, the Toronto Alexithymia Scale (TAS-20), and the Distress Disclosure Index (DDI). Latent profile analysis was employed to identify distinct alexithymia profiles. Multivariate logistic regression was used to analyze sociodemographic variables within each profile, and ANOVA was used to examine relationships between alexithymia profiles and self-disclosure.

**Results:**

Alexithymia in female patients with PFD can be categorized into three profiles: low-level alexithymia group(*n* = 87, 19.3%), the moderate alexithymia group(*n* = 309, 68.7%), and high-level alexithymia group(*n* = 54, 12.0%). Multivariate logistic regression revealed that age, educational level, per capita monthly household income, and duration of illness were significantly associated with membership in alexithymia profiles. Total self-disclosure scores different significant across the three alexithymia profiles among female patients with PFD (*P* < 0.05).

**Conclusion:**

Alexithymia in female patients with PFD can be categorized into three profiles, with significant differences in self-disclosure levels across these subgroups. Assessing alexithymia may help identify high-risk patients with insufficient self-disclosure and provide a basis for stratified, individualized support for emotional expression and communication. Future longitudinal studies are needed to clarify further the temporal and causal relationships between alexithymia and self-disclosure.

## 1 Introduction

Pelvic floor dysfunction (PFD) encompasses a group of chronic conditions resulting from damage to the pelvic floor supportive structures, functional decline, or abnormalities in neuromuscular regulation. It predominantly affects females, with primary clinical manifestations including stress urinary incontinence, pelvic organ prolapse, chronic pelvic pain, sexual dysfunction, and fecal incontinence (1). It is prevalent in females (2).As the population ages and fertility patterns change, the prevalence of PFD continues to rise, with a trend toward younger patients. As the population ages and fertility patterns change, the prevalence of PFD continues to rise, with a trend toward younger patients (3). Research indicates that the global prevalence of PFD among adult females is approximately 20% to 40% (4), while in China, it ranges from 40% to 60.2% (5). It is projected that by 2050, the number of females affected by PFD worldwide will increase to 43.8 million (6). PFD imposes a substantial burden on global public health and presents a growing societal challenge. Therefore, increasing attention to this patient population is of crucial importance (7).

Despite continuous advances in the diagnosis, treatment, and rehabilitation of PFD, the condition is characterized by a protracted course and recurrent symptoms, involving highly private domains such as urinary or fecal leakage, pelvic organ prolapse, and sexual dysfunction (8). These symptoms not only restrict patients’ daily activities and physical function but may also trigger feelings of shame, stigma, and body image disturbance (9), further increasing the risk of psychosocial issues, including anxiety, depression, social withdrawal, and impaired intimate relationships (10). Influenced by the disease’s private nature and sociocultural norms, some patients tend to conceal symptoms, suppress or avoid expressing negative emotions, leading to alexithymia, which ultimately affects their proactive help-seeking, access to social support, and disease self-management (11). Therefore, health management for female PFD patients should not be limited to pelvic floor function and physical symptoms but should also address their emotional well-being (12).

Alexithymia is a relatively stable personality trait characterized by difficulties in identifying and describing emotions and by externally oriented thinking (13). Studies have shown that the prevalence of alexithymia in female patients with PFD can reach 53.3% (14). Individuals with high levels of alexithymia, when facing disease-related stress, often struggle to identify and express their own emotions accurately, and tend to focus on external events or somatic sensations rather than internal emotional experiences (15). For female PFD patients, this trait may further hinder their ability to express feelings and needs to partners, family members, and healthcare providers, affecting their psychological adaptation and, delaying the disease recovery process, and reducing treatment adherence (16). Previous research has indicated that alexithymia is closely associated with adverse psychological outcomes such as anxiety and depression (17). Therefore, identifying the distinct manifestations of alexithymia in female PFD patients is of great significance for developing targeted psychological nursing strategies.

With the development of positive psychology, research has gradually shifted from focusing on disease-related negative emotions and psychological disorders to exploring positive psychological factors that help patients adapt to illness, mobilize psychosocial resources, and improve health outcomes (18). Self-disclosure is the process by which individuals actively express their experiences, thoughts, and emotions to others and is an important pathway for promoting emotional regulation, acquiring social support, and effective communication (19). For female patients with PFD, the privacy of the disease and potential stigma may reduce their expression of illness experiences and emotional feelings, thereby limiting opportunities to obtain emotional support and professional help (20). In contrast, high levels of self-disclosure help improve doctor-patient communication, promote resource acquisition, and enhance psychological adaptability (21). From an emotional processing perspective, alexithymia and self-disclosure are intrinsically linked; if individuals have difficulty accurately identifying and describing their own emotions, their ability to convert internal experiences into language and express them externally may also be constrained (22). Previous studies have found an association between self-disclosure and alexithymia in patients with coronary heart disease (23) and elderly HIV/AIDS patients (24). Still, this relationship remains insufficiently studied in female patients with PFD.

Importantly, alexithymia is not a unidimensional psychological symptom but rather a complex, subjective experience encompassing physiological, emotional, and cognitive domains (25). Traditional research has predominantly relied on total scores from alexithymia scales to assess severity, which may obscure clinically meaningful heterogeneity in its expression across individuals. In recent years, there has been growing interest in using model-based classification methods, such as latent profile analysis (LPA) (26), to identify subtypes of alexithymia. For example, Yan et al. (27) used LPA to classify alexithymia in patients undergoing *in vitro* fertilization–embryo transfer into three subgroups—low-level, moderate, and high-level alexithymia—and successfully analyzed differences in clinical characteristics and modifiable factors across these subgroups, which informed the design of personalized interventions. Similarly, Wang et al. (28) used LPA to examine the relationship between anxiety, depressive symptoms, and alexithymia among Chinese nursing students, identifying three subgroups and distinguishing them based on baseline sociodemographic variables. Taken together, these studies indicate that person-centered analytical methods can identify subgroups with distinct symptom profiles, potentially supporting stratified and personalized intervention approaches.

However, current research on alexithymia in the field of female PFD remains insufficient. First, previous studies have largely evaluated alexithymia as a homogeneous construct, with limited attention to distinct symptom combinations and potential heterogeneity among patients; it remains unclear whether female PFD patients exhibit distinct latent subgroups of alexithymia with different characteristics. Second, evidence is also lacking on whether different latent subgroups of alexithymia demonstrate varying levels of self-disclosure. Therefore, this study aims to use LPA to identify latent classes of alexithymia among female PFD patients and to compare self-disclosure levels across these classes, thereby providing a theoretical basis for developing stratified, individualized intervention strategies for emotional expression and self-disclosure.

## 2 Objects and methods

### 2.1 Design and procedure

This study employed a convenience sampling method to recruit 450 female patients with PFD who sought treatment at the Gynecology and Urologic Pelvic Floor Comprehensive Treatment Unit of a Grade A Level 3 hospital in Urumqi, Xinjiang, China, between December 2025 and April 2026.Before recruitment, the purpose of the study, survey content, and procedures were thoroughly explained to all potential participants, who were informed of their right to withdraw at any time. This study was conducted in accordance with the principles of the Declaration of Helsinki and was approved by the Ethics Committee of the First Affiliated Hospital of Xinjiang Medical University (Approval No.: K202511-33).

### 2.2 Participants

The inclusion criteria were as follows (1): meeting the diagnostic criteria for PFD (29); (2) age ≥ 18 years; and (3) informed and willing to participate, having signed a written informed consent form. The exclusion criteria were as follows: (1) cognitive impairment or altered consciousness; (2) presence of severe complications or other serious systemic diseases; (3) a documented diagnosis of mental disorders (e.g., depression, anxiety disorder, schizophrenia) by a qualified mental health professional, as self-reported via a brief pre-survey health screening questionnaire; (4) pregnancy, lactation, or postoperative recovery (less than 3 months post-surgery); and (5) experience of a major negative life event within the past 6 months.

The sample size for quantitative data was calculated using the formula (30): *N = Z²_1-α/2_P* (1 − *P*)/*d*². At a 95% confidence interval, *Z*_1-α/2_ = 1.96; *d* represents the absolute error or accuracy rate, which is 0.05 in this study; *P* is the prevalence of alexithymia among female PFD patients, and based on data from previous studies (14), *P* is 53.3%. According to this formula, the theoretical sample size was 383. Considering a 15% invalid questionnaire rate, the planned sample size is at least 451 cases, and the final sample size obtained is 465 cases.

### 2.3 Measurement instruments

#### 2.3.1 Demographic and clinical characteristics

A self-designed questionnaire was developed based on a literature review and the input of two clinical pelvic floor specialists. The questionnaire included age, educational level, marital status, occupation, place of residence, personality type, average monthly household income, insurance, healthcare payment methods, number and method of deliveries, type of disease, duration of illness, and whether the patient had received pelvic floor rehabilitation treatment.

#### 2.3.2 Toronto Alexithymia Scale

The Toronto Alexithymia Scale (TAS-20) was used to assess alexithymia levels among participants. Originally developed by Taylor et al. (31) and subsequently localized into Chinese and revised by Yi Jinyao (32). The scale consists of 20 items three dimensions: difficulty identifying feelings (7 items), difficulty describing feelings (5 items), and externally oriented thinking (8 items)—for a total of 20 items. Each item uses a 5-point Likert scale, ranging from 1 (“Strongly Disagree”) to 5 (“Strongly Agree”). Items 4, 5, 10, 18, and 19 are reverse-scored before calculating the total score, which ranges from 20 to 100 points. A total score of 61 or higher indicates the presence of alexithymia; higher scores indicate greater severity. In this study, the Cronbach’s α for this scale was 0.820, indicating good reliability.

#### 2.3.3 Distress disclosure index

The Distress Disclosure Index (DDI) was used to assess individuals’ willingness and tendency to disclose personal experiences of distress and other private issues to others. Originally developed by Kahn et al. (33) and later adapted into Chinese and revised by Li Xinmin (34). The scale is unidimensional and consists of 12 items. Each item uses a 5-point Likert scale, ranging from 1 (“Strongly Disagree”) to 5 (“Strongly Agree”). Items 2, 4, 5, 8, 9, and 10 are reverse-scored before calculating the total score, which ranges from 12 to 60 points; higher scores indicate greater self-disclosure of inner thoughts. In this study, the scale’s Cronbach’s α was 0.854.

### 2.4 Data collection and quality control

This study is a cross-sectional survey that used a questionnaire to collect data. Before the survey, the researchers underwent relevant training and obtained approval from the appropriate hospital departments. Members of the research team explained the purpose and significance of the study to the participants, as well as instructions for completing the questionnaire. Each questionnaire took approximately 30 minutes to complete. Participants filled out the questionnaires on-site, and researchers collected them immediately, checking for any omissions or missing information. Within 24 hours of the survey’s completion, two investigators cross-checked the raw data, and data management was conducted through independent dual data entry, logical verification, and outlier review.

### 2.5 Data analysis

Data analysis was conducted using SPSS 27.0 and Mplus 8.3. First, a latent profile model was estimated using the mean scores across the three alexithymia dimensions. Model fit was evaluated using the Akaike Information Criterion (AIC), the Bayesian Information Criterion (BIC), and the sample-adjusted Bayesian Information Criterion (aBIC); lower values indicate better model fit. Entropy was used to evaluate classification accuracy, with higher values indicating greater accuracy. The Lo-Mendell-Rubin likelihood ratio test (LMRT) and the bootstrapped likelihood ratio test (BLRT) were used to compare models with different numbers of classes; a lower p-value indicates that a k-class model provides a better fit than a k-1-class model. In addition to fit indices, model parsimony (favoring simpler models) and class size (at least 5% of the total sample to ensure replicability) were considered when selecting the optimal model. The final number of profiles was determined based on theoretical relevance and practical significance to ensure that the chosen model provided meaningful insights for the research objectives. *P* < 0.05 was considered statistically significant.

SPSS 27.0 software was used for data entry and descriptive statistical analysis. Quantitative data conforming to a normal distribution were expressed as mean ± standard deviation ( $\bar{x}$ ± s), and categorical variables were expressed as frequency and percentage (*n*, %). Intergroup comparisons were conducted using the chi-square test or the Kruskal–Wallis H test. Statistically significant variables from univariate analysis were entered into a multinomial logistic regression model to examine independent factors associated with the attribution of alexithymia in female patients with PFD. Additionally, one-way analysis of variance (ANOVA) was used to examine differences in general demographic data and self-disclosure scores across latent-class patients. If the overall differences were statistically significant, pairwise comparisons were further conducted using the LSD or Bonferroni method. All tests were two-sided, and the significance level α was set at 0.05.

## 3 Results

### 3.1 Common method bias

To assess potential common method bias in this study, the Harman single-factor test was employed. An exploratory factor analysis was conducted on all items of the Toronto Alexithymia Scale and the Distress Disclosure Index, extracting nine common factors with eigenvalues greater than 1. Among these, the first factor accounted for 22.11% of the variance, which did not exceed the critical threshold of 40%. This indicates that the data in this study were not significantly affected by common method bias.

### 3.2 Demographic and clinical disease characteristics

In this study, a total of 465 questionnaires were collected. Among these, 7 questionnaires showed a clear pattern in their responses, and 8 questionnaires were incompletely filled out and thus excluded. Ultimately, 450 questionnaires were retrieved, resulting in a recovery rate of 96.8%. Among the valid respondents (*n* = 450*)*, the average age was 53.75 ± 13.14 years. Among them, 295 patients were over 50 years old. In terms of disease types, there were 168 cases of stress urinary incontinence, 151 cases of pelvic organ prolapse, and 108 cases of chronic pelvic pain. Surgical treatment was received by 216 cases, and pelvic floor rehabilitation therapy by 234 cases.

### 3.3 Latent profile determination

LPA was used to assess alexithymia in 450 female patients with PFD, using “difficulty identifying feelings,” “difficulty describing feelings,” and “externally oriented thinking” as observational indicators, and fitting 1- to 5-class models sequentially (**Table 1**). The results showed that as the number of model classes increased, the AIC, BIC, and aBIC values gradually decreased. For the 3-class model, both the LMRT and BLRT tests were statistically significant (*P* < 0.05), and the entropy value was 0.887 (> 0.80), indicating high classification accuracy. In the 4-class and 5-class models, the LMRT was not significant (*P* > 0.05). Therefore, after careful consideration, the 3-class model was selected as the optimal solution. To verify the reliability of the classification results, the average assignment probabilities for each latent class were calculated, yielding values of 92.9%, 93.4%, and 97.0%, respectively (all > 90%).

**Table 1:** Results of potential profiles of alexithymia (*n =* 450).

Model	AIC	BIC	aBIC	LMR(*P*)	BLRT(*P*)	Entrop	Proportions(%)
1	6792.010	6816.665	6797.623	−	−	−	−
2	6522.390	6563.482	6531.746	0.023	<0.001	0.719	0.720/0.280
3	6291.041	6348.570	6304.140	<0.001	<0.001	0.887	0.193/0.687/0.120
4	6271.648	6345.614	6288.489	0.055	<0.001	0.731	0.282/0.122/0.118/0.478
5	6254.333	6344.936	6275.117	0.147	<0.001	0.770	0.124/0.451/0.300/0.066/0.059

AIC, Alike Information Criterion; BIC, Bayesian Information Criterion; aBIC, Adjusted BIC; LMR, Lo-Mendell-Rubin; BLRT, Bootstrap Likelihood Ratio Test;-, not applicable.

### 3.4 Characteristics and naming of latent profiles

**Figure 1:**
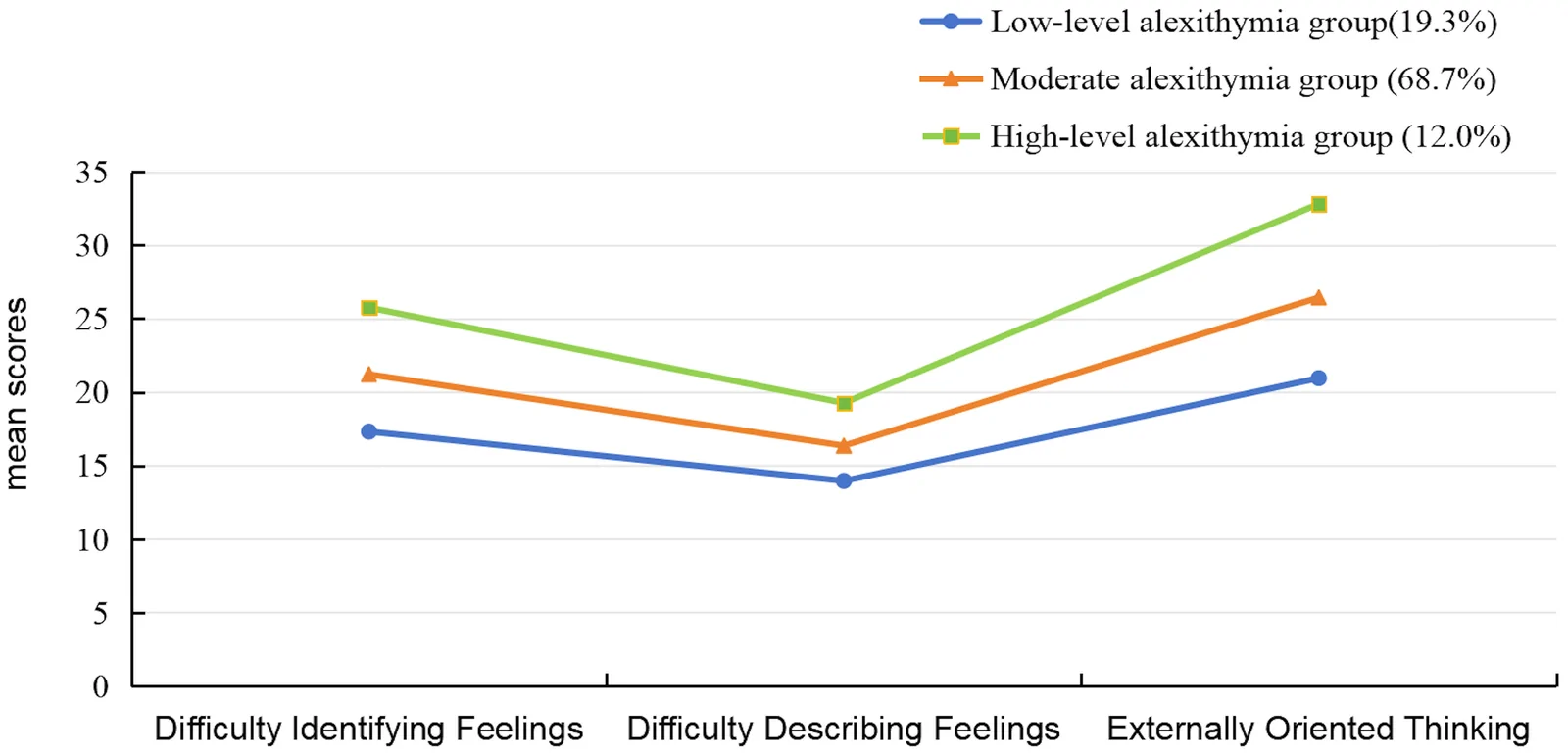
Mean scores of dimensions for alexithymia profiles.

The scores for the three latent profiles of alexithymia in female patients with PFD, across the dimensions of difficulty identifying feelings, difficulty describing feelings, and externally oriented thinking, are presented in **Figure 1**. The score trends across profiles were generally consistent. Profile 1 included 87 patients (19.3%) with an alexithymia score of 52.08 ± 3.72, whose scores across all three dimensions were relatively low, with the lowest in difficulty describing feelings. This profile was therefore labeled as the “Low-level alexithymia group.” Profile 2 comprised 309 patients (68.7%) with an alexithymia score of 64.19 ± 3.58, whose scores across all alexithymia dimensions were moderate. Accordingly, this group was labeled as the “Moderate alexithymia group.” Profile 3 consisted of 54 patients (12.0%), with an alexithymia score of 78.33 ± 2.69, who exhibited high scores across all three dimensions, with the highest score in alexithymia. This group was thus labeled as the “High-level alexithymia group.” Analysis of variance showed that the differences among groups in difficulty identifying feelings, difficulty describing feelings, externally oriented thinking, and the total alexithymia score were statistically significant (all *P*<0.001); see **Table 2**. Furthermore, difficulty identifying feelings, difficulty describing feelings, and externally oriented thinking were all positively correlated (*r* = 0.577–0.636; all *P* < 0.001). The effect size for the externally oriented thinking dimension was the largest (η² = 0.670), indicating that this dimension made the most significant contribution to distinguishing between different alexithymia classes.

**Table 2:** Comparison of alexithymia scores among female PFD patients with different potential profiles (*n=* 450).

Profiles	Difficulty identifying feelings	Difficulty describing feelings	Externally oriented thinking	Alexithymia total scores
Profile 1	17.32 ± 1.94	13.89 ± 1.28	20.87 ± 2.50	52.08 ± 3.72
Profile 2	21.26 ± 1.87	16.40 ± 1.56	26.52 ± 2.36	64.19 ± 3.58
Profile 3	25.91 ± 1.76	19.35 ± 1.86	33.07 ± 2.11	78.33 ± 2.69
*F*	356.563	210.952	454.148	945.652
*P*	<0.001	<0.001	<0.001	<0.001

Profile1, Low-level alexithymia group; Profile2, Moderate alexithymia group; Profile3, High-level alexithymia group.

### 3.5 Univariate analysis of factors associated with alexithymia profiles

Univariate analysis revealed no statistically significant differences among the three latent classes of female PFD patients in terms of marital status, occupation, place of residence, personality type, method of medical payment, number of deliveries, mode of delivery, type of disease, and treatment method (all *P* > 0.05). In contrast, significant differences were observed in age, educational level, average monthly household income, and duration of illness (all *P* < 0.05). These differences are shown in **Table 3**.

**Table 3:** Univariate analysis of alexithymia among female patients with PFD [n(%)].

Variables	Total(*n* = 450)	Profile1:Low-level alexithymia group (*n*= 87)	Profile 2:Moderate alexithymia group (*n* = 309)	Profile 3:High-level alexithymia group(*n* = 54)	X^2^/*H*	*P*
Age(years)					15.184^a^	0.004
18-35	53 (11.78)	20 (22.99)	28 (9.06)	5 (9.26)		
36-50	102 (22.66)	13 (14.94)	78 (25.24)	11 (20.37)		
>50	295 (65.56)	54 (62.07)	203 (65.70)	38 (70.37)		
Education level					25.950^a^	<0.001
Junior high school and below	209 (46.44)	29 (33.33)	145 (46.93)	35 (64.81)		
Technical secondary school/High school	124 (27.56)	19 (21.84)	93 (30.10)	12 (22.22)		
College and above	117 (26.00)	39 (44.83)	71 (22.97)	7 (12.96)		
Marital status					0.557^b^	0.757
Unmarried	25 (5.56)	4 (4.60)	18 (5.83)	3 (5.56)		
Married	416 (92.44)	81 (93.10)	286 (92.55)	49 (90.74)		
Divorced/widowed	9 (2.00)	2 (2.30)	5 (1.62)	2 (3.70)		
Occupation					0.437^b^	0.804
Student	10 (2.22)	0 (0.00)	9 (2.91)	1 (1.85)		
Farmer	39 (8.67)	5 (5.75)	28 (9.06)	6 (11.11)		
Employed	275 (61.11)	59 (67.82)	183 (59.22)	33 (61.11)		
Retirement	126 (28.00)	23 (26.43)	89 (28.81)	14 (25.93)		
Residence					0.970^a^	0.616
Rural	151 (33.56)	29 (33.33)	107 (34.63)	15 (27.78)		
City	299 (66.44)	58 (66.67)	202 (65.37)	39 (72.22)		
Personality Types					6.270^a^	0.180
Introverted	163 (36.22)	33 (37.93)	109 (35.28)	21 (38.89)		
Extroverted	143 (31.78)	24 (27.59)	96 (31.07)	23 (42.59)		
Mixed	144 (32.00)	30 (34.48)	104 (33.65)	10 (18.52)		
per capita monthly household income(yuan)					16.12^2)^	0.013
<2000	56 (12.45)	5 (5.75)	38 (12.30)	13 (24.07)		
2000~4000	227 (50.44)	43 (49.43)	160 (51.78)	24 (44.45)		
4001~6000	113 (25.11)	22 (25.28)	81 (26.21)	10 (18.52)		
>6000	54 (12.00)	17 (19.54)	30 (9.71)	7 (12.96)		
Healthcare payment methods					3.565^a^	0.168
Employee medical insurance	205 (45.56)	37 (42.53)	137 (44.34)	31 (57.41)		
Self-pay medical care	245 (56.44)	50(57.47)	172 (55.66)	23 (42.59)		
Number of deliveries					1.751^a^	0.782
0	41 (9.11)	6(6.90)	30 (9.71)	5 (9.26)		
1	134 (29.78)	24 (27.58)	96 (31.07)	14 (25.93)		
>2	275 (61.11)	57 (65.52)	183 (59.22)	35 (64.81)		
Mode of delivery					3.233^a^	0.520
No childbirth	41 (9.11)	6(6.90)	30 (9.71)	5 (9.26)		
Vaginal delivery	360 (80.00)	74 (85.05)	243(78.64)	43 (79.63)		
Cesarean section	49 (10.89)	7(8.05)	36(11.65)	6 (11.11)		
Disease types					0.306^b^	0.858
Stress Urinary Incontinence	168 (37.33)	31 (35.63)	117 (37.86)	20 (37.04)		
Pelvic Organ Prolapse	151 (33.56)	36 (41.38)	98 (31.72)	17 (31.48)		
Chronic Pelvic Pain	108 (24.00)	17 (19.54)	77 (24.92)	14 (25.93)		
Sexual Dysfunction	15 (3.33)	2 (2.30)	11 (3.56)	2 (3.70)		
Fecal Incontinence	8 (1.78)	1 (1.15)	6 (1.94)	1 (1.85)		
Duration of illness(years)					11.092^a^	0.026
<1	167 (37.11)	41 (47.13)	113 (36.57)	13 (24.08)		
1~2	74 (16.45)	10 (11.49)	57 (18.45)	7 (12.96)		
>2	209 (46.44)	36 (41.38)	139 (44.98)	34 (62.96)		
Treatment method					1.306^a^	0.521
Surgical treatment	216 (48.00)	37 (42.53)	152 (49.19)	27 (50.00)		
Pelvic floor rehabilitation	234(52.00)	50(57.47)	157(50.81)	27(50.00)		

a is the X^2^ value; b is the Kruskal-Wallis H Test value.

### 3.6 Multivariate logistic regression of factors associated with alexithymia profiles

A multivariate logistic regression analysis was conducted, using four univariately significant factors as independent variables and the three alexithymia latent profiles in female PFD patients as the dependent variable, with profile 1, “Low-level alexithymia group,” as the reference group. The results indicated that age, level of education, monthly per capita income, and duration of illness were significantly associated with membership in latent profiles (*P* < 0.05). Compared with patients with junior high school and below, those with college and above were classified into profile 2 (OR = 0.400, *P* = 0.004) and profile 3 (OR = 0.158, *P* < 0.001), with a lower probability than patients in profile 1. Regarding age, compared with patients aged 18–35 years, those aged 36–50 years were more likely to be classified into profile 2 (OR = 3.769, *P* = 0.002). Regarding per capita monthly household income, compared with those earning less than 2,000 yuan, patients earning 2,000–4,000 yuan were less likely to be classified into profile 3 (OR = 0.222, *P* = 0.013). Additionally, patients with a disease duration of more than 2 years (OR = 2.490, *P* = 0.029) were more likely to be classified into profile 3 than profile 1. **Table 4** provides a detailed account of the findings.

**Table 4:** The multivariate logistic regression analysis of latent profiles of alexithymia in female patients with PFD (*n* = 450).

Dependent variable	Independent variables	Reference	B	OR	*P*	95%*CI*
Profile2: Moderate alexithymia group vs Profile1: Low-level alexithymia group	Intercept		1.344	3.835	0.030	1.139~12.917
	Age (years)	18~35				
	36~50		1.327	3.769	0.002	1.609~8.828
	>50		0.639	1.895	0.072	0.944~3.806
	Education level	Junior high school and below				
	Technical secondary school/High school		-0.028	0.973	0.933	0.507~1.865
	College and above		-0.917	0.400	0.004	0.216~0.741
	per capita monthly household income (yuan)	<2000				
	2000~4000		-0.750	0.472	0.147	0.171~1.302
	4001~6000		-0.482	0.618	0.382	0.210~1.819
	>6000		-0.989	0.372	0.098	0.115~1.198
	Duration of illness (years)	<1				
	1~2		0.653	1.922	0.107	0.868~4.254
	>2		0.250	1.284	0.367	0.746~2.209
Profile 3: High-level alexithymia group vs Profile1: Low-level alexithymia group	Intercept		0.287	1.333	0.731	0.260~6.837
	Age (years)	18~35				
	36~50		1.089	2.972	0.107	0.790~11.182
	>50		0.415	1.514	0.479	0.481~4.764
	Education level	Junior high school and below				
	Technical secondary school/High school		-0.546	0.579	0.236	0.235~1.428
	College and above		-1.848	0.158	<0.001	0.057~0.439
	per capita monthly household income(yuan)	<2000				
	2000~4000		-1.503	0.222	0.013	0.068~0.725
	4001~6000		-1.263	0.283	0.062	0.075~1.064
	>6000		-0.962	0.382	0.195	0.089~1.636
	Duration of illness (years)	<1				
	1~2		0.655	1.924	0.282	0.584~6.340
	>2		0.912	2.490	0.029	1.097~5.656

The low-level alexithymia group is the reference category.

### 3.7 Comparison of self-disclosure scores among female PFD patients with different latent profiles of alexithymia

The ANOVA showed statistically significant differences in self-disclosure scores among patients with different alexithymia profiles (*F* = 38.176, *P* < 0.001). *Post hoc* pairwise comparisons using the Bonferroni correction revealed that the moderate alexithymia group scored higher than the high-level alexithymia group, while the low-level alexithymia group scored higher than both the high and moderate alexithymia groups; all differences were statistically significant (all *P* < 0.05). The results suggest that the lower a patient’s alexithymia, the higher their self-disclosure. **Table 5** provides a detailed account of the findings.

**Table 5:** Comparison of self-disclosure among different alexithymia groups in female patients with PFD (*n* = 450).

Variables	*n*	Self-disclosure scores(Mean ± SD)
Low-level alexithymia group	87	36.47 ± 6.66
Moderate alexithymia group	309	33.01 ± 4.25^*^
High-level alexithymia group	54	29.06 ± 5.44^*^^▲^
*F*		38.176
*P*		<0.001

Compared with the low-level alexithymia group, *P < 0.05; compared with the moderate alexithymia group, ▲*P* < 0.05.

## 4 Discussion

Three distinct latent profiles of alexithymia were identified among female patients with PFD. The study found that factors such as age, educational level, per capita monthly household income, and duration of illness may influence these profiles. Additionally, comparisons of alexithymia profile characteristics revealed significant differences in self-disclosure scores.

### 4.1 Heterogeneity of alexithymia in female patients with PFD

The results of this study indicate that alexithymia in female patients with PFD can be categorized into three distinct latent profiles, with significant individual differences. Among them, the low-level alexithymia group accounted for 19.3% of the total sample and scored the lowest on the difficulty-in-expressing-emotions dimension, indicating relatively mild overall alexithymia. Although some patients may still exhibit deficits in emotion identification or externally oriented thinking, they may possess relatively better emotional awareness, emotion-related cognitive processing, and self-expression abilities (28). Therefore, for this subgroup of patients, healthcare providers should attend to their potential emotion-regulation needs and recommend preventive education, including information about alexithymia and instruction in emotion-expression techniques.

The moderate alexithymia group constituted the largest proportion, accounting for 68.7% of patients, indicated by high difficulty identifying feelings and externally oriented thinking. This suggests it is the dominant alexithymia feature among female PFD patients, consistent with findings reported by Wang Yanbo et al. (35). This distribution may be attributed to the demographic characteristics of the study population, which is predominantly middle-aged and older. Patients in this age group may be deeply influenced by traditional sociocultural norms and family roles, leading them to feel confused about their emotions and to find it difficult to identify them (36). Consequently, they may perceive it as challenging to describe their feelings to others in appropriate language. This difficulty may drive them to adopt avoidant approaches in interpersonal relationships, thereby preventing embarrassment stemming from poor emotional processing and limited expressive ability. Such behavior may result in a loss of intimacy while simultaneously diminishing self-esteem and self-worth (37). For this subgroup of patients, it is recommended that healthcare providers strengthen the assessment of emotional identification and expression abilities, and through emotion recognition training, supportive communication, and appropriate family involvement, help patients accurately identify and express their feelings, improve emotional communication and social support, thereby promoting their psychosocial adaptation.

The high-level alexithymia group accounted for 12.0%. Individuals in this group showed elevated scores across all three dimensions, exhibiting prominent alexithymia features. Even when they have some awareness of their internal emotional states, they often fail to express specific feelings accurately and tend to attribute emotional distress to external events or somatic symptoms rather than describe emotional experiences to others (38). For this population, it is recommended that healthcare providers focus on identifying risk factors and screening high-risk individuals. These patients should be guided to distinguish emotional somatization from disease-related symptoms, and timely intervention may improve disease management and mental health outcomes.

It is worth noting that the primary distinguishing feature of the three profiles identified in this study lies in the overall high or low scores across all dimensions, rather than a qualitative pattern of high scores in one dimension and low scores in another across the three dimensions of alexithymia. In other words, these three groups of patients exhibit a gradient in the overall severity of alexithymia, rather than qualitative differences in configuration between subtypes. This finding suggests that alexithymia in female PFD patients may be better understood from the perspective of a severity continuum. At the same time, this may corroborate the methodological value of using LPA to stratify patients from an individual-centered perspective, providing an empirical foundation for future studies that incorporate additional clinical indicators to explore potential qualitative subtypes.

### 4.2 Analysis of factors influencing the latent profiles of alexithymia in female patients with PFD

The study results indicate that compared with younger patients, middle-aged female PFD patients are more likely to be classified into the “moderate alexithymia group.” This may be attributed to the fact that women in this age group often bear the dual burden of family and career, where multiple role conflicts tend to lead them to neglect their own health and delay seeking medical care. Prolonged disease-related distress not only depletes emotional regulation resources but may also impair emotional recognition and expression abilities (39). Additionally, the decline in estrogen levels during the perimenopausal period may trigger negative experiences such as sleep disturbances and mood instability, further exacerbating emotional distress (40). Therefore, it is recommended that healthcare providers pay attention to the psychological and emotional well-being of patients in this age group, extending beyond physical symptom assessment to incorporate perimenopausal health education, pelvic floor rehabilitation guidance, and mindfulness-based emotional awareness training, while also guiding patients to express disease-related concerns, which may help reduce alexithymia levels.

The study results showed that patients with lower educational levels were more likely to be classified into the “moderate alexithymia group” and “high-level alexithymia group.” The reason may be that female PFD patients with lower educational levels have certain limitations in the acquisition, comprehension, and application of health information, and have relatively insufficient awareness of pelvic floor diseases and related symptoms. Meanwhile, their social interactions and social support resources may be relatively limited, thereby increasing the difficulty of identifying, understanding, and accurately expressing their own emotional experiences (41). Therefore, it is recommended that healthcare professionals adopt plain, specific, and easy-to-understand language for patients with lower educational levels or limited ability to comprehend health information, and use demonstrations, pictures, and other methods to help them understand information about pelvic floor disease. Additionally, open-ended questioning and guided communication should be used to encourage patients to describe their own feelings, thereby improving their emotional recognition and expression abilities.

The findings indicated that patients with lower average household income were more likely to belong to the “high-level alexithymia group.” This may be because the prolonged course of PFD requires some patients to use long-term care supplies, and the resulting sustained medical expenses could exacerbate the financial burden on low-income patients (42). Additionally, repeated medical visits and rehabilitation treatments may disrupt patients’ normal work schedules, interrupt their work rhythm, and reduce their income sources (43). Furthermore, low-income individuals often face difficulties in accessing medical services, psychosocial support, and rehabilitation resources, potentially leading to insufficient professional guidance and social support, which may ultimately worsen alexithymia (44). Therefore, it is recommended that healthcare providers assess economic status during treatment and provide information on financial assistance programs, unemployment benefits, and medical policies. Governments should allocate resources effectively, strengthen medical insurance coverage for the disease, and offer vocational rehabilitation guidance to help patients return to the workforce, thereby alleviating financial burdens and potentially reducing the risk of alexithymia.

The results showed that patients with longer disease duration may be classified into the “high-level alexithymia group.” PFD is characterized by chronic persistence and a tendency to recur. With prolonged disease duration, the cumulative effect of symptoms and repeated healthcare-seeking experiences may induce learned helplessness, undermine treatment confidence, and promote the formation of negative emotional coping patterns (45). Meanwhile, long-term illness may restrict patients’ social participation, reduce opportunities for interpersonal interaction and expression, and cause negative emotions to accumulate persistently without effective release (46). Therefore, it is recommended that healthcare providers include patients with longer disease duration as key targets in psychological follow-up management, through disease trajectory education, shared decision-making, and staged goal setting, to help them rebuild a sense of control, reduce disease progression, and potentially alleviate alexithymia.

### 4.3 Variation in self-disclosure scores among patients with different alexithymia profiles

This study revealed significant differences in self-disclosure among three potential categories of alexithymia. Analysis of variance showed that patients in the “low-level alexithymia group” exhibited significantly higher self-disclosure than those in the “moderate alexithymia group” and the “high-level alexithymia group.” These findings are similar to those of Zhang et al. (23) in a study of nursing students. According to emotion regulation theory, self-disclosure is an important means by which individuals regulate negative emotions, and it can help them recognize and reappraise stressful events and their effects through processes such as cognitive reappraisal (47). The results of this study suggest that higher levels of alexithymia may be associated with lower levels of self-disclosure, which may be related to patients’ difficulties in emotional identification, understanding, and expression. Due to difficulty accurately perceiving and describing their own emotional experiences, such patients may face obstacles in communicating disease-related feelings and seeking social support (48). At the same time, the tendency of patients with alexithymia to focus on somatic sensations, along with accompanying psychological characteristics such as anxiety, depression, and social avoidance, may be related to their reduced self-disclosure. Furthermore, PFD involves private health issues such as urination, defecation, and sexual function, and patients may reduce communication about related experiences due to feelings of shame, stigmatization, and concerns about negative evaluation (49). When patients fail to receive adequate understanding and positive responses from partners or healthcare providers, their communication of disease-related information and emotional exchange may be affected to some extent. Therefore, it is recommended that healthcare providers prioritize assessing alexithymia in female patients with PFD and address patients’ needs across different levels of alexithymia regarding emotional identification and disease communication. Through emotional recognition and expression training, supportive communication, and the creation of a safe, non-judgmental communication environment, patients may be better able to express their experiences of disease and psychological feelings, thereby promoting the use of social support.

### 4.4 Limitations and outlook

This study still has certain limitations. First, alexithymia was primarily assessed using self-report scales, which may be influenced by social expectations, recall bias, and individual self-awareness. Future studies could combine clinical interviews, raters’ assessments, and multi-source data to improve the accuracy and comprehensiveness of alexithymia assessment. Second, this study employed single-center convenience sampling, with participants drawn from female PFD patients at a single hospital; thus, the representativeness of the sample and the generalizability of the findings may be limited. Future studies should conduct multi-regional, multicenter, large-sample studies using more representative sampling methods to further validate the stability of the potential categorical structure and the generalizability of the research conclusions. Third, the confounding factors included in this study primarily focused on sociodemographic and disease-related characteristics and did not yet cover key psychosocial factors that may influence the formation of latent categories of alexithymia. Future research could further incorporate variables such as anxiety, depression, coping strategies, social support, severity of pelvic floor symptoms, and quality of life to explore in depth the associated factors and mechanisms of action for different categories of alexithymia. It is worth noting that the latent categories identified in this study primarily manifest as a gradient of severity in alexithymia; no configurational subgroups with distinct qualitative characteristics were identified. This may be related to the limited number of indicators, the high correlation among the three dimensions of the TAS-20, and insufficient heterogeneity in the single-center sample. Future research could incorporate additional emotion-cognition-related measures and broaden the sample’s clinical and demographic heterogeneity to further explore distinct subtypes of alexithyia. Finally, because this study employed a cross-sectional design, it could only reflect the characteristics of the relationship between alexithymia and self-disclosure at a specific point in time; it was unable to infer causal relationships among variables or to examine dynamic changes within potential categories. Future studies could adopt longitudinal and intervention-based designs to further track the evolution of potential alexithymia categories and validate the effectiveness of stratified care interventions based on distinct psychological characteristics.

## 5 Conclusion

In summary, from an individual-differences perspective, this study employed LPA to explore the heterogeneity of alexithymia among female patients with PFD and identified three latent classes with distinct features: low, moderate, and high. Significant differences were observed among patients across latent classes of alexithymia in sociodemographic and disease-related characteristics, with age, education level, per capita monthly household income, and duration of illness potentially associated with latent class membership. Notably, patients in the low-level alexithymia group exhibited higher levels of self-disclosure, suggesting that distinct latent features of alexithymia may relate to patients’ self-disclosure. Healthcare providers should prioritize screening for self-disclosure levels in the “high-level alexithymia group,” as this group demonstrated relatively lower self-disclosure. Future research may consider developing and testing intervention strategies tailored to different alexithymia profiles to enhance self-disclosure and promote well-being. However, such approaches require validation through longitudinal or experimental studies before they can be translated into clinical practice.

## Data Availability

The raw data supporting the conclusions of this article will be made available by the authors, without undue reservation.
